# TRAIL is involved in CpG ODN-mediated anti-apoptotic signals

**DOI:** 10.3892/or.2011.1579

**Published:** 2011-12-06

**Authors:** EUN-JUNG LIM, DAE-WEON PARK, TAE-WHAL JEONG, BYUNG-RHO CHIN, YOE-SIK BAE, SUK-HWAN BAEK

**Affiliations:** 1 Department of Biochemistry and Molecular Biology, Aging-Associated Vascular Disease Research Center, College of Medicine, Yeungnam University, Daegu; 2 Department of Dentistry, College of Medicine, Yeungnam University, Daegu; 3 Department of Biological Sciences, Sungkyunkwan University, Suwon, Republic of Korea

**Keywords:** CpG ODN, FoxO3a, TRAIL, anti-apoptosis

## Abstract

Synthetic oligodeoxynucleotides (ODNs) with the CpG-motifs are recognized by toll-like receptor 9 (TLR9), which elicits an immune response. Serum starvation of Raw264.7 cells increased tumor necrosis factor-related apoptosis-inducing ligand (TRAIL) expression. However, treatment with CpG ODN reduced TRAIL expression as well as apoptosis by serum starvation. In serum starved cells, TLR9 inhibitors recovered the decreasing TRAIL expression and sub-G1 accumulation by CpG ODN. CpG ODN-regulated anti-apoptotic signals which were dependent on the Akt-FoxO3a signaling pathway. CpG ODNs activated Akt and inactivated FoxO3a in serum starved cells. Knockdown of FoxO3a by siRNA decreased TRAIL expression and apoptosis in serum-starved cells. In contrast, FoxO3a overexpression increased apoptosis by serum starvation, and CpG ODNs blocked these effects through TRAIL expression. LY294002, a PI3K-Akt inhibitor, blocked the CpG ODN effect of TRAIL expression and the sub-G1 population in serum starved cells. In contrast, overexpression of wild-type Akt reduced additional sub-G1 cells both in non-CpG ODN- and CpG ODN-treated cells. Taken together, these results demonstrate the involvement of Akt-FoxO3a signaling in TLR9-mediated downregulation of TRAIL and anti-apoptotic signals.

## Introduction

Toll-like receptors (TLRs) recognize a set of conserved molecular structures called pathogen-associated molecular patterns, which allow them to sense innate and adaptive immune responses. Among them, TLR9 is essential for recognition of microbial CpG DNA or synthetic CpG oligonucleotide analogs containing a CpG oligodeoxynucleotide (ODN). CpG DNA activates macrophages, monocytes, and dendritic cells to secrete proinflammatory cytokines (1,2). The binding of CpG DNA to TLR9 and subsequent endosomal maturation are thought to be essential for CpG DNA-driven immunostimulatory activity (3). After CpG DNA binding, TLR9 signaling is initiated by recruitment of the adaptor molecule MyD88 followed by the engagement of interleukin (IL)-1R-associated kinases and tumor necrosis factor (TNF)-α receptor (TNFR)-associated factor 6 (4). These complexes activate the IκB kinase complex and subsequently activate NF-κB-dependent pro-inflammatory cytokines such as TNF-α and IL-1β (5). TLRs are members of the IL-1R superfamily and share a common activation pathway through their Toll/IL-1R signaling domain (6). Despite this common pathway, TLRs show differences in their rate, intensity, or efficiency of activation by yet unidentified mechanisms.

Members of the FoxO subfamily of forkhead transcription factors include the mammalian ortholog DAF-16, which regulates longevity in the nematode *Caenorhabditis elegans* (7). Mice and humans possess three highly related FoxO homologs (FoxO1, FoxO3 and FoxO4) with overlapping expression patterns and transcriptional activities (8). Suppression of FoxO transcriptional activity by Akt-mediated phosphorylation leads to enhanced cell survival (9). In conditions in which the Akt survival and growth pathway is activated, FoxO3a is phosphorylated by Akt and exported to the cytoplasm (10). In contrast, unphosphorylated FoxO3a proteins are active forms and are located in the nucleus where they bind to their target gene promoters. Overexpression of the constitutively activated form of FoxO3a leads to apoptosis in many cell types (11). Additionally, FoxO3a mediates apoptosis by activating pro-apoptotic genes such as TNF-related apoptosis-inducing ligand (TRAIL) (12). Although FoxO3a has generally been considered an inducer of apoptosis, there is little evidence of TLR signaling.

In this study, we investigated the role of TRAIL in TLR9-mediated anti-apoptosis of macrophages. We found that CpG ODN treatment blocked serum deprivation-mediated apoptosis. We also found that CpG ODN downregulated TRAIL gene expression. We further investigated the mechanisms of CpG ODN-induced TRAIL expression via the TLR9-Akt-FoxO3a signaling pathway.

## Materials and methods

### Reagents and antibodies

Cell culture reagents were obtained from Life Technologies (Grand Island, NY, USA). Fetal bovine serum (FBS) was obtained from Thermo Scientific HyClone (Logan, UT). Chloroquine, propidium iodide (PI) and β-actin antibody were obtained from Sigma-Aldrich (St. Louis, MO, USA). Phosphorothioated unmethylated endotoxin-free CpG ODN (B-class, TCCATGA**CG**TTCCTGATGCT) and control ODN 1720 (TCCATGAGCTTCCTGATGCT, inactive control for CpG ODN 1668) were obtained from Genotech (Daejeon, South Korea), and an RNA reverse transcription-polymerase chain reaction (RT-PCR) core kit was purchased from Axygen Biosciences (Union City, CA, USA). Antibodies (Abs) against FoxO3a and Akt were purchased from Cell Signaling Technology (Beverly, MA, USA). Bafilomycin A1 and LY294002 were purchased from Calbiochem (San Diego, CA, USA).

### Cell culture

The Raw264.7 macrophage cell line was obtained from the American Type Culture Collection (Manassas, VA, USA). Cells were grown in Dulbecco's modified Eagle's medium (Invitrogen, Carlsbad, CA, USA) containing 10% FBS, 2 μM L-glutamine, 10 U/ml penicillin and 10 μg/ml streptomycin at 37°C in a humidified atmosphere under 5% CO_2_. Cells were treated with synthetic CpG ODN for various times.

### Fluorescence-activated cell sorting (FACS) analysis

To quantify apoptotic nuclei, cells were fixed in ethanol, stained with 50 μg/ml PI and RNase A for 30 min at room temperature followed by washing, and the samples were processed by flow cytometry using a FACSCalibur apparatus (BD Biosciences, Franklin Lakes, NJ, USA). The results are shown as a histogram with sub-G1 positive cells considered the apoptotic cells.

### Western blot analysis

The cells were washed with cold-PBS, trypsinized, and pelleted at 700 × g. Cell pellets were resuspended in lysis buffer comprised of 50 mM Tris-HCl (pH 8.0), 5 mM EDTA, 150 mM NaCl, 0.5% Nonidet P-40, 1 mM PMSF, and a protease inhibitor cocktail. The preparations were then cleared by centrifugation, and the supernatants were saved as cell lysates. Proteins were separated by 8% reducing sodium dodecyl sulfate-polyacrylamide gel electrophoresis and immunoblotted in 20% methanol, 25 mM Tris, and 192 mM glycine onto nitrocellulose membranes. The membranes were then blocked with 5% non-fat dry milk in TTBS (25 mM Tris-HCl, 150 mM NaCl, and 0.2% Tween-20) and incubated with primary Ab for 4 h. Subsequently, membranes were washed, incubated for 1 h with secondary Ab conjugated to horseradish peroxidase, rewashed, and finally developed using an enhanced chemiluminescence system (Amersham, Buckinghamshire, UK).

### Real-time RT-PCR

Total RNA was extracted from cells using TRIzol reagent (Invitrogen). Total RNA (1 μg) was used as a template to make first strand cDNA by oligo-dT priming using a reverse transcriptase system (Promega, Madison, WI, USA). Real-time RT-PCR was performed using a LightCycler 1.5 (Roche Diagnostics, Almere, The Netherlands) with SYBR-Green I as the florescent dye, according to the manufacturer's instructions. The synthetic gene-specific primer sets used for PCR were: i) TRAIL forward primer, 5′-CCTCTCGGAAAGGGCATTC-3′, and reverse primer, 5′-TCCTGCTCGATGACCAGCT-3′, which amplified 70 bp of the mouse TRAIL cDNA; ii) β-actin forward primer, 5′-AGAGGGAAATCGTGCGTGAC-3′, and reverse primer, 5′-CAATAGTGATGACCTGGCCGT-3′, which amplified 137 bp of the mouse β-actin cDNA. Cycling conditions were 95°C for 10 min, followed by 45 cycles of 95°C for 10 sec, 62°C for 5 sec, and 72°C for 6 sec. Target genes were normalized to β-actin for quantification.

### Knock-down of FoxO3a using small interfering RNA (siRNA)

Oligonucleotides corresponding to the mouse FoxO3a siRNA sequence 5′-UGAUGAUCCACCAAGAGCUCUUGCC-3′ were purchased from Invitrogen. A control siRNA was also purchased and used. For transfection, 2×10^6^ Raw264.7 cells were resuspended in a nucleoporator buffer (Lonza, Allendale, NJ, USA) with 200 pmole siRNA. Cells were nucleoporated according to the manufacturer's protocol, and the above genes were knocked down for 24 h.

### FoxO3a and Akt overexpression

A vector encoding a FoxO3a protein (pLenti6/V5-D-TOPO-FoxO3a) was generously provided by Dr Kim (Yeungnam University, South Korea) for cell protein expression. Akt was subcloned into the pEGFP-C1 mammalian expression vector. Cells were transfected with the control vector, wild-type FoxO3a, or Akt cDNA for 24 h, and fresh medium was added. Cells transfected with the cDNA were cloned by serial dilution in a 96-well plate in a culture medium with selecting antibiotics to obtain a stable cell line. Sub-culturing was continued for 4 weeks, and then wells representing a single colony were selected and expression was confirmed using the protein level as determined by Western blot analysis.

## Results

### CpG ODN blocks TRAIL expression and apoptosis using serum starvation

**Figure 1:**
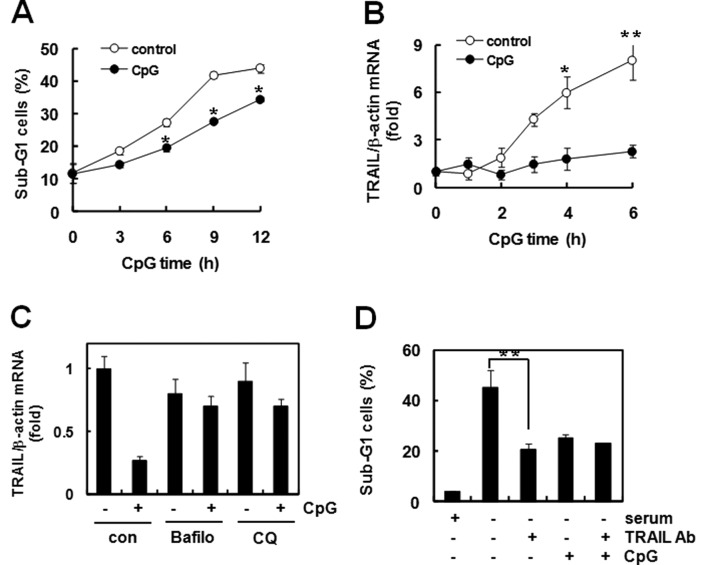
Synthetic oligodeoxynucleotides (ODN) with the CpG-motif (CpG ODN) inhibit apoptosis induced by serum starvation by downregulating tumor necrosis factor-related apoptosis-inducing ligand (TRAIL) in macrophages. (A) Raw264.7 cells were cultured, changed to serum-free medium, and incubated with medium (control) or 3 μM CpG ODN (CpG) for the indicated times. Cells were stained with propidium iodide (PI) and analyzed for apoptosis by flow cytometry. (B) Cultured cells were changed to serum-free medium and incubated with 3 μM CpG ODN for the indicated times. Total RNA was then isolated from the cells and subjected to real-time RT-PCR. The graph represents the fold-changes in TRAIL mRNA after the cells were treated with 3 μM CpG ODN. Each data point represents the mean ± SD of three independent experiments (^*^P<0.05). (C) Cultured cells were changed to serum free medium and pretreated with 5 nM bafilomycin A1 or 5 μg/ml CQ prior to CpG ODN stimulation for 6 h. mRNA levels were analyzed by real-time PCR using specific TRAIL or β-actin primers. (D) Cells were pretreated with neutralizing TRAIL antibody (Ab), CpG ODN, or a combination of TRAIL Ab plus CpG ODN in serum-free medium for 12 h. The cells were stained with PI and analyzed for the induction of apoptosis by flow cytometry. Each data point represents the mean ± SD of three independent experiments (^**^P<0.01).

We first investigated the effect of CpG ODN in serum starvation-induced apoptosis. As expected, CpG ODN had a significant inhibitory effect on sub-G1 cell accumulation following serum starvation (Fig. 1A). The change of TRAIL mRNA expression using serum starvation in Raw264.7 cells was further examined. While serum starvation induced a dramatic increase in TRAIL expression in a time-dependent manner, CpG ODN strongly inhibited this response (Fig. 1B). We studied whether TLR9 mediates the inhibition of TRAIL expression and apoptosis. Pretreatment with bafilomycin A1 or chloroquine recovered TRAIL expression reduced by CpG ODN (Fig. 1C). To confirm the role of TRAIL in apoptosis following serum starvation, we treated Raw264.7 cells with the neutralizing TRAIL antibody or a combination with CpG ODN. Apoptosis partially but significantly decreased in the TRAIL neutralizing antibody-treated cells, and this effect was very similar to that of the CpG ODN treated cells. However, no additional effect was observed with the combination of CpG ODN and the TRAIL neutralizing antibody (Fig. 1D). These results suggest that CpG ODN plays a role in serum starvation-induced apoptosis by inhibiting TRAIL expression.

### FoxO3a is involved in CpG ODN-regulated TRAIL expression

**Figure 2:**
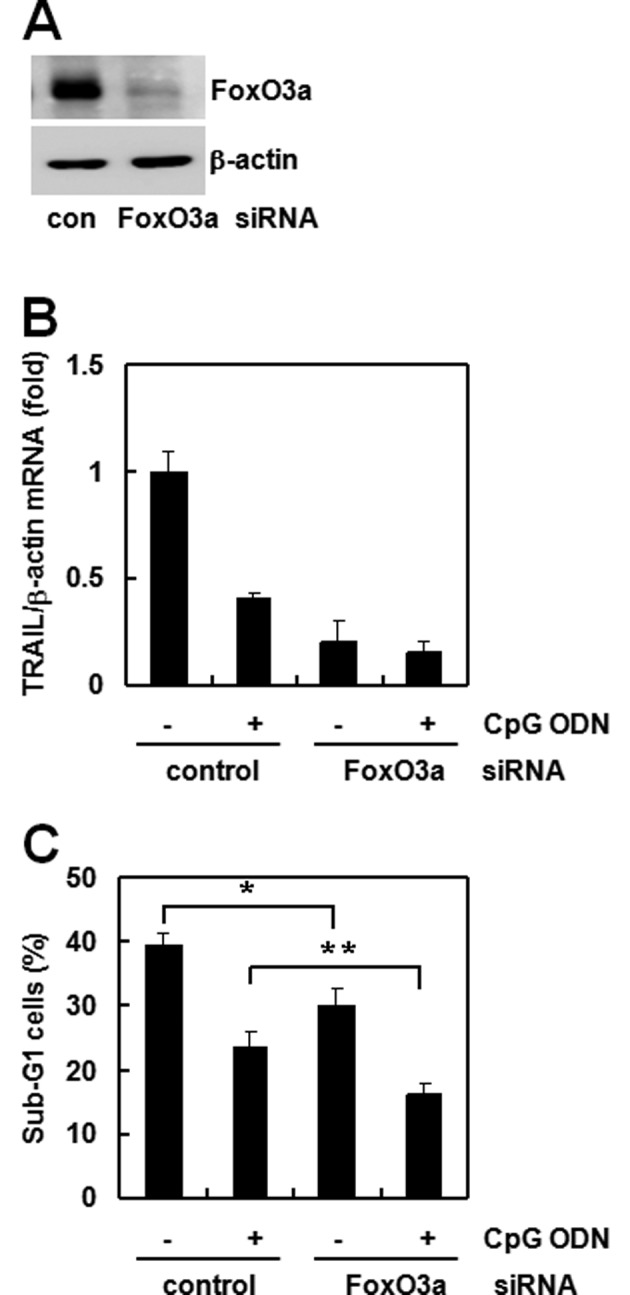
FoxO3a siRNA decreases tumor necrosis factor-related apoptosis-inducing ligand (TRAIL) expression. Cells were transfected with control siRNA or FoxO3a siRNA. (A) FoxO3a expression was assessed by Western blotting using anti-FoxO3a antibody. (B) Control or FoxO3a siRNA cells were changed to serum free medium. After 9 h, the cells were incubated with synthetic oligodeoxynucleotides (ODN) with the CpG-motif (CpG ODN) for 6 h, and TRAIL mRNA levels were assessed by real-time PCR. (C) siRNA cells were changed to serum-free media and treated with 3 μM CpG ODN for 12 h, stained with propidium iodide (PI), and analyzed for the induction of apoptosis by flow cytometry. Each data point represents the mean ± SD of four independent experiments (^*^P<0.05 and ^**^P<0.01).

**Figure 3:**
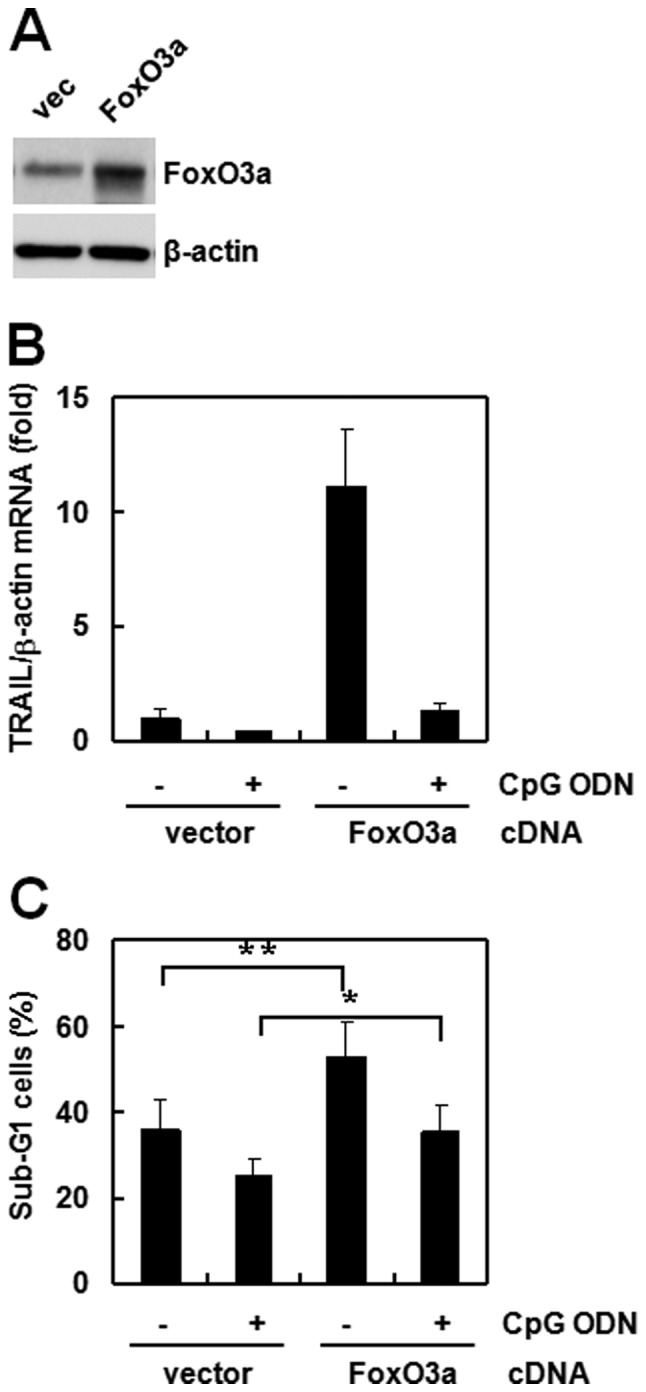
FoxO3a overexpression increases tumor necrosis factor-related apoptosis-inducing ligand (TRAIL) expression. Cells were stably transfected with a control vector (pLenti/V5-D-TOPO) or cDNA encoding wild-type FoxO3a. (A) FoxO3a expression was assessed by Western blotting using anti-FoxO3a Ab. (B) Control vector or FoxO3a overexpressing cells were changed to serum-free medium. After 9 h, cells were incubated with synthetic oligodeoxynucleotides (ODN) with the CpG-motif (CpG ODN) for 6 h, and TRAIL mRNA levels were assessed by real-time PCR. (C) Vector or FoxO3a overexpressing cells were changed to serum free media and treated with 3 μM CpG ODN for 12 h, stained with propidium iodide (PI), and analyzed for the induction of apoptosis by flow cytometry. Each data point represents the mean ± SD of three independent experiments (^*^P<0.05 and ^**^P<0.01).

FoxO3a is a well known transcription factor for TRAIL regulation (13). Therefore, we assessed the involvement of FoxO3a in CpG ODN-mediated TRAIL downregulation using siRNA or an overexpression technique. Transfection of FoxO3a siRNA effectively decreased protein expression in Raw264.7 cells (Fig. 2A). Then, we compared the CpG ODN effect between control and FoxO3a siRNA cells. TRAIL expression was reduced dramatically in FoxO3a siRNA cells compared to that of control siRNA cells. Treatment with CpG ODN reduced TRAIL expression (Fig. 2B) and sub-G1 accumulation in both siRNA cells (Fig. 2C). To confirm the role of FoxO3a, we overexpressed the gene in the same cell lines and investigated the effect of CpG ODN. TRAIL expression increased strongly in FoxO3a overexpressed cells compared to that of empty-vector transfected cells (Fig. 3A). However, CpG ODN treatment decreased TRAIL expression (Fig. 3B) and sub-G1 accumulation (Fig. 3C) in both types of transfected cells. These results suggest that FoxO3a directly regulates TRAIL expression and that the anti-apoptosis effect of CpG ODN occurs through TRAIL.

### The Akt-FoxO3a pathway is involved in regulating TRAIL expression and apoptosis

**Figure 4:**
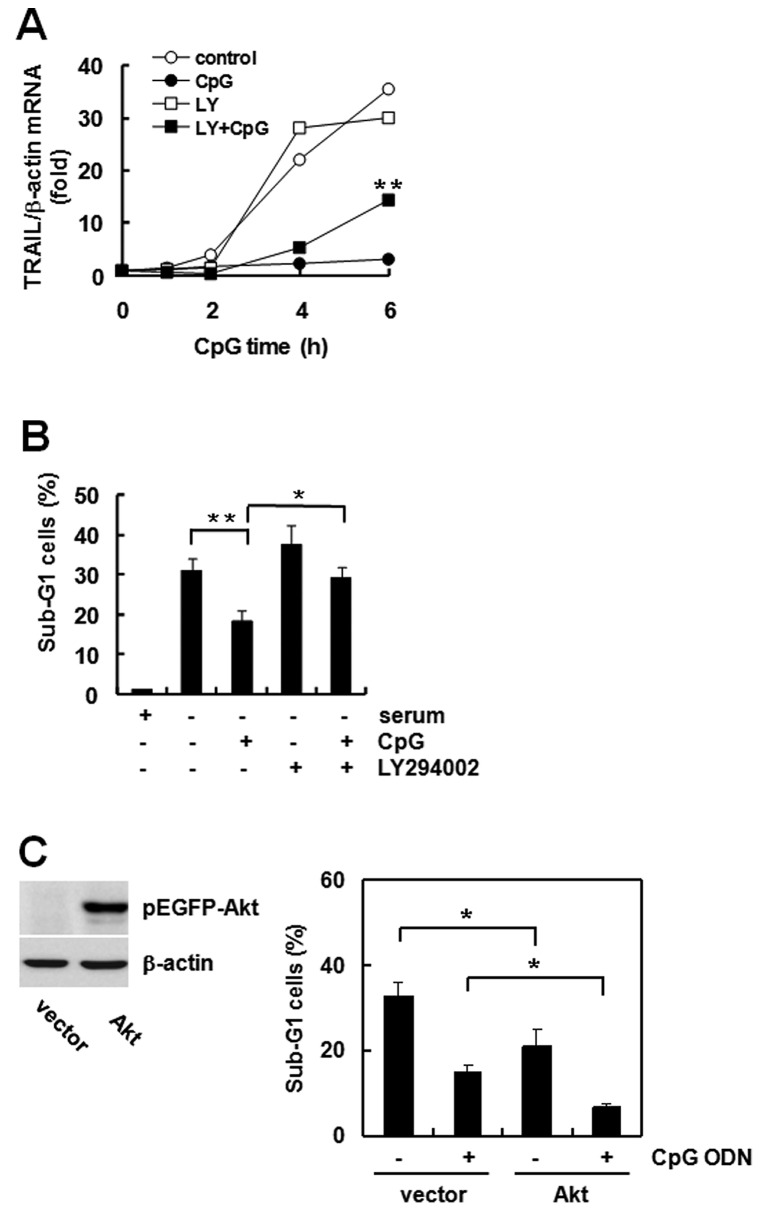
The Akt pathway is involved in synthetic oligodeoxynucleotides (ODN) with the CpG-motif (CpG ODN)-mediated anti-apoptosis through tumor necrosis factor-related apoptosis-inducing ligand (TRAIL) expression. (A) Cells were serum starved for 12 h, pretreated with 10 μl LY294002, and incubated with 3 μM CpG ODN for the indicated times. TRAIL mRNA levels were assessed by real-time PCR. (B) Cells were changed to serum free medium, pretreated with 10 μM LY294002, and incubated with 3 μM CpG ODN for 12 h. Cells were stained with propidium iodide (PI), and analyzed for the induction of apoptosis by flow cytometry. Each data point represents the mean ± SD of four independent experiments (^*^P<0.05 and ^**^P<0.01). (C) Cells were transiently transfected with a control vector (pEGFP) or cDNA encoding wild-type Akt. Akt expression was assessed by Western blotting using anti-Akt antibody. Vector or Akt overexpressing cells were changed to serum-free medium and treated with 3 μM CpG ODN for 12 h, stained with PI, and analyzed for the induction of apoptosis by flow cytometry. Each data point represents the mean ± SD of four independent experiments (^*^P<0.05).

Akt is a well known apoptosis regulatory kinase, and FoxO3a phosphorylation by Akt leads to inactivation of its transcriptional activity (14). Previously, we observed that CpG ODN phosphorylates Akt for activation and phosphorylates FoxO3a for inactivation (15). Therefore, we investigated the role of Akt in TRAIL expression and apoptosis by CpG ODN. While CpG ODN blocked serum starvation-induced TRAIL expression, LY294002 pretreatment partially and significantly recovered the reduced TRAIL expression (Fig. 4A). Apoptosis increased following LY294002 treatment (Fig. 4B). To confirm the role of Akt in apoptosis, we transfected the GFP vector or GFP-tagged Akt cDNA and compared the effect of CpG ODN. Akt expression increased in Akt cDNA transfected cells. Akt transfection itself decreased the sub-G1 population compared to that of vector-transfected cells, and the decrease was deepened by CpG ODN (Fig. 4C). These results suggest that Akt is a very important regulator of CpG ODN-mediated TRAIL expression and anti-apoptosis.

## Discussion

TLRs including TLR9 play a central role in innate immunity by mediating pathogen recognition (16). Previous studies have demonstrated a role for TLR9 in mediating the effects of cell survival including macrophages. We also observed that treating macrophages with TLR9 agonists strongly obviated apoptosis. Many groups have investigated the regulatory proteins involved in cell survival, but the specific proteins involved in the response to a TLR9 agonist have not been clarified. CpG ODN promotes cell survival via Hsp70 upregulation to increase Bcl-xL (17). Furthermore, Hsp90β is also involved in the TLR9 anti-apoptotic effect (18). Therefore, we examined the effect of a TLR9 agonist on the cellular levels of the many proteins participating in apoptotic pathways. Among the possible regulatory factors, our results identified TRAIL as an important regulator of macrophages in apoptosis.

Our results demonstrate that CpG ODN strongly downregulated TRAIL expression in macrophages via the TLR9-dependent pathway. TRAIL is involved in apoptosis signaling pathways, specifically by modulating immune system function (19). Interestingly, TRAIL receptor-mediated apoptosis is inhibited by FLIP, through suppression of either recruitment of procaspase-8 by FADD or autocatalytic activation of caspase-8 (20,21). Therefore, our results suggest that TRAIL is suppressed after CpG ODN treatment, and that these responses could interfere with serum deprivation-induced apoptosis.

NF-κB is a transcription factor that potentially affects the expression of many genes and may favor cell survival by upregulating gene products with anti-apoptotic properties or downregulating pro-apoptotic factors (22). It has been suggested that activating NF-κB via the TLR2 signaling pathway and the subsequent induction of gene expression can protect cells from FasL-induced apoptosis (23). Recognition of TLR9 by CpG ODN is also followed by NF-κB activation (3). Despite this, the expression of several apoptosis-regulating genes is controlled by other transcription factors including FoxO (24).

Previous studies have suggested that activating Akt negatively regulates FoxO transcription factors (9,25), and that the direct phosphorylation of Akt inhibits FoxO3a transcriptional activation (26). Additionally, the PI3K/Akt pathway is very important for the anti-apoptosis effects of CpG ODN (27). Furthermore, because Akt activity prevents the induction of apoptosis by cytokines, growth factors, and cellular stress (28), we determined the effect of the Akt pathway on FoxO3a activation, TRAIL expression, and apoptosis. Use of the pharmacological inhibitor LY294002 enabled us to assess the role of the Akt signaling pathway in the regulation of FoxO3a phosphorylation. Our data show that inhibiting Akt resulted in a significant increase in TRAIL gene expression in these cells. Furthermore, direct evidence was obtained by wild-type FoxO3a overexpression or using siRNA. Our data demonstrate that CpG ODN treatment increased TRAIL expression in FoxO3a overexpressing cells compared to vector control cells. FoxO3a siRNA cells demonstrated an opposite result compared to that of overexpressing cells. Together, these data strongly support the conclusion that FoxO3a is a transcription factor for TRAIL regulation in response to CpG ODN.

CpG ODN protects B-cells and macrophages against apoptosis (17,29). Based on this result, we confirmed that engaging TLR9 protected against serum deprivation-induced apoptosis. Furthermore, we showed that CpG ODN induced an increase in FoxO3a phosphorylation. This protective effect was controlled by decreased TRAIL expression in CpG ODN-stimulated macrophages. The anti-apoptotic effects of CpG ODN stimulation require participation of the Akt-FoxO3a signaling pathway. Taken together, these results suggest that TLR9 triggers the FoxO3a transcription factor through Akt signaling, and that the regulation of TRAIL by CpG ODN may contribute to the anti-apoptotic effect.

## Abbreviations

TRAIL: tumor necrosis factor-related apoptosis-inducing ligand
CpG ODN: CpG oligodeoxynucleotide
TLR9: toll-like receptor 9
FoxO: forkhead transcription factors of the O class
